# Neutrophil Crawling in Capillaries; A Novel Immune Response to *Staphylococcus aureus*

**DOI:** 10.1371/journal.ppat.1004379

**Published:** 2014-10-09

**Authors:** Mark Geoffrey Harding, Kunyan Zhang, John Conly, Paul Kubes

**Affiliations:** 1 The Calvin, Phoebe, and Joan Snyder Institute for Chronic Diseases, University of Calgary, Calgary, Alberta, Canada; 2 Department of Microbiology, Immunology and Infectious Diseases, Cumming School of Medicine, University of Calgary, Calgary, Alberta, Canada; 3 Department of Medicine, Cumming School of Medicine, University of Calgary, Calgary, Alberta, Canada; 4 Department of Pathology and Laboratory Medicine, University of Calgary, Calgary, Alberta, Canada; 5 Department of Physiology and Pharmacology, University of Calgary, Calgary, Alberta, Canada; 6 Department of Critical Care, Cumming School of Medicine, University of Calgary, Calgary, Alberta, Canada; National Institutes of Health, United States of America

## Abstract

Methicillin-resistant *Staphylococcus aureus* (MRSA), particularly the USA300 strain, is a highly virulent pathogen responsible for an increasing number of skin and soft tissue infections globally. Furthermore, MRSA-induced soft tissue infections can rapidly progress into life-threatening conditions, such as sepsis and necrotizing fasciitis. The importance of neutrophils in these devastating soft tissue infections remains ambiguous, partly because of our incomplete understanding of their behaviour. Spinning disk confocal microscopy was used to visualize the behaviour of GR1-labelled neutrophils in subcutaneous tissue in response to GFP-expressing MRSA attached to a foreign particle (agarose bead). We observed significant directional neutrophil recruitment towards the *S. aureus* agarose bead but not a control agarose bead. A significant increase in neutrophil crawling within the capillaries surrounding the infectious nidus was noted, with impaired capillary perfusion in these vessels and increased parenchymal cell death. No neutrophils were able to emigrate from capillaries. The crawling within these capillaries was mediated by the β_2_ and α_4_ integrins and blocking these integrins 2 hours post infection eliminated neutrophil crawling, improved capillary perfusion, reduced cell death and reduced lesion size. Blocking prior to infection increased pathology. Neutrophil crawling within capillaries during MRSA soft tissue infections, while potentially contributing to walling off or preventing early dissemination of the pathogen, resulted in impaired perfusion and increased tissue injury with time.

## Author Summary

Methicillin-resistant *Staphylococcus aureus* (MRSA) is a highly virulent pathogen responsible for a significant portion of skin and soft tissue infections throughout the world. We investigated the role of neutrophils in soft tissue infections, as these immune cells have been shown to be both essential for clearance of this pathogen but also for increasing tissue injury associated with *S. aureus* infections. We visualized the behaviour of neutrophils in the subcutaneous tissue following the introduction of a localized infectious stimulus. In addition to a profound neutrophil recruitment into the infectious nidus, significant neutrophil crawling in capillaries surrounding the region was also noted, a region of vasculature which has not previously been associated with neutrophil recruitment during infection. The neutrophils were not seen to emigrate from the capillaries but rather were retained in these vessels and maintained a crawling behaviour via β_2_ and α_4_ integrins. Blocking these integrins released the neutrophils from the capillaries, reinstituted capillary perfusion, and reduced the surrounding cell death leading to reduced lesion size following infection. Neutrophil crawling within capillaries during MRSA soft tissue infections, while potentially contributing to walling off or preventing dissemination of the pathogen, resulted in impaired perfusion and increased tissue injury.

## Introduction

*Staphylococcus aureus* is a Gram-positive, facultatively anaerobic bacterium that poses considerable challenges to human health as a re-emerging pathogen in both hospital and community settings. As a commensal bacterium, approximately 50% of the general population carry *S. aureus* in the anterior nares [1]. Despite its commensal status, *S. aureus* is a serious pathogen, responsible for approximately 18,500 deaths per year in the United States, more than all deaths caused by AIDS, influenza, or viral hepatitis [2]. *S. aureus* infections, particularly those due to methicillin-resistant Staphylococcus aureus (MRSA) have been increasing in frequency in recent years, and now account for the majority of all clinical skin and soft tissue infections in the United States [3]. Importantly, these infections can cause serious complications, such as necrotizing fasciitis, necrotizing pneumonitis and sepsis [4]. A single MRSA strain, pulsotype USA300 is the dominant community acquired strain in North America [5], [6], [7], [8].

During *S. aureus* soft tissue infections, pattern recognition receptors such as NOD2 and TLR2, as well as complement fragments, induce signalling pathways that promote neutrophil recruitment critical for abscess formation and clearance of the bacteria [9]. The importance of neutrophils in *S. aureus* infections cannot be understated; neutrophils are the first to arrive at the local infectious nidus, migrate out of the vasculature, and attempt to eradicate the pathogen through an armamentarium of defenses that include oxidant production, as well as the release of proteases, defensins and various other toxins [10], [11]. Neutropenia leads to uncontrolled infection in mice, impaired healing, and increased likelihood of *S. aureus* dissemination that can lead to sepsis [12]. Additionally, neutrophil deficiencies (either genetic, or due to treatments such as chemotherapy or corticosteroids) make individuals highly susceptible to infection with *S. aureus* [11]. Paradoxically, these same defenses so critical to survival can also injure host tissues [13]–[15]. In fact, delayed neutropenia can actually provide some benefit to tissue repair associated with *S. aureus* soft tissue infections. Additionally, *S. aureus* can survive when phagocytosed by neutrophils [16] and the neutrophil may act as a “Trojan horse”, allowing the bacteria to disseminate from the point of infection and cause additional damage to the host [17]. Therefore, early neutrophil recruitment is critical to protect the host from the bacterial infection, but later neutrophil recruitment leads to additional bystander tissue damage, and may actually be a mechanism by which *S. aureus* enhances its virulence [18].

Neutrophil recruitment to a site of infection occurs exclusively from the post-capillary venules with no published reports of recruitment from other vascular structures such as arterioles or capillaries. The first step of the cascade subcutaneously is tethering and rolling, mediated largely by P- and E-selectin on endothelial cells binding with P-selectin glycoprotein ligand-1 (PSGL-1) on neutrophils [19]. This is followed by firm adhesion to the endothelium, typically mediated by the integrin LFA-1 [20]. Neutrophils then crawl inside the vessel, migrating along the vessel wall, usually perpendicular to or against blood flow via Mac-1 [20]. Although α_4_β_1_ (VLA-4) has also been reported to have a minor contribution in mouse neutrophils [21], in humans it appears to be upregulated and contributes primarily in severe infections such as sepsis [22]. Following adhesion and crawling, neutrophils emigrate predominantly via a junctional, paracellular pathway or at times transcellularly using integrins and intracellular adhesion molecules (ICAMs). In addition, platelet/endothelial cell adhesion molecule (PECAM-1, also known as CD31), junctional adhesion proteins (JAMs), and endothelial cell selective adhesion molecules (ESAM) play important roles in neutrophil emigration from the vasculature [23], [24].

This study made use of spinning disk intravital microscopy to visualize the behaviour of neutrophils in the first few hours following a localized nidus of *S. aureus* infection, introduced here as a small foreign inert particle, an agarose bead. The use of the agarose beads ensured that each mouse received a limited amount of bacteria to a very localized area that would best mimic the most common cause of *S. aureus* skin and soft tissue infection, namely a post puncture wound localized soft tissue infection. The approach unveiled a novel mechanism of neutrophil crawling within capillaries that we had not observed previously with intradermal injection of vast amounts of *S. aureus* disseminated over large areas of tissue. The random back and forth crawling of neutrophils within capillaries around the nidus of infection may occur in an attempt to prevent dissemination of bacteria through these vessels or to reduce pH in the area. Because we identified the molecular mechanisms of this capillary walk, we were able to inhibit this phenomenon and noted that this may also contribute to impaired capillary perfusion, increased cell death and increased lesion size of the classical open wound noted in patients with *S. aureus* infection.

## Results

### Model Development

**Figure 1:**
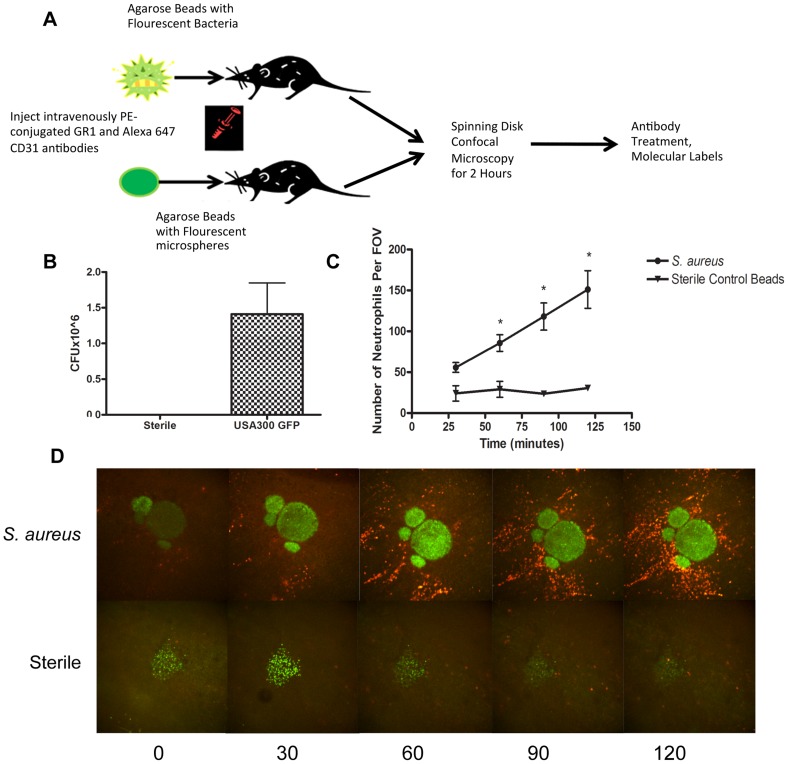
Parameters for *S. aureus* infection. 1a: Flowchart of *S. aureus* bead model skin preparation. Mice were anesthetized, and then either an agarose bead with GFP-expressing *S. aureus* or an agarose bead with GFP fluorescent microspheres was inserted into the subcutaneous tissue below the superficial fascia. The mice were then treated IV anti-GR1 antibodies conjugated to PE (to enable neutrophil detection). Mice were then imaged for 2 hours at 4× magnification, and then treated with blocking antibodies to interrogate different aspects of the model system. 1b: CFU used for bead generation. N = 10 independent experiments for the GFP-expressing USA300 strain, and 3 for sterile microspheres. 1c: The number of neutrophils being recruited to an *S. aureus* bead over two hours. The number of neutrophils was quantified at 30, 60, 90 and 120 minutes post-insertion of the bead into the skin tissue. N = 4 for control beads, and N = 11 for *S. aureus* beads. * *p*<0.05. 1d: Representative images of neutrophil recruitment to a bead with GFP-expressing *S. aureus* (above) and with microspheres (below). Images were taken at 30, 60, 90 and 120 minutes after insertion of the bead into the subcutaneous skin tissue. The neutrophils are shown in red, labelled with anti-Ly6g. In green (above) is the bead with GFP-expressing *S. aureus*, and below, the green is the sterile microspheres.

In order to visualize using spinning disk confocal microscopy, neutrophil recruitment to a localized *S. aureus* infection as might happen following a puncture wound with secondary infection, an agarose bead was inserted into the subcutaneous tissue layer, beneath the connective tissue of the skin via a fine needle to deliver a very small and reproducible amount of bacteria. The infected bead was easily visualized, due to the green fluorescent protein (GFP)-expressing bacteria and the sterile bead was visualized due to fluorescence nanoparticles (Figure 1a). This permitted us to examine the entire process of immune cell recruitment into the infected site, allowing for effective localization of the pathogen, and clear visualization of changes in neutrophil behaviour over time. Addition of the foreign particle with *S. aureus* was important as this has been shown to be more pathogenic [25]–[27], thus requiring fewer colony-forming units (CFUs) to induce an infection, and permitted optimal modelling of neutrophil responses to MRSA [28]. Since the size of bead linearly predicted the amount of bacteria delivered, we used beads 250–350 µm in diameter that delivered ∼10^6^ bacteria. Sterile beads used as negative controls had no CFUs (Fig. 1b).

### Neutrophil Recruitment

**Figure 2:**
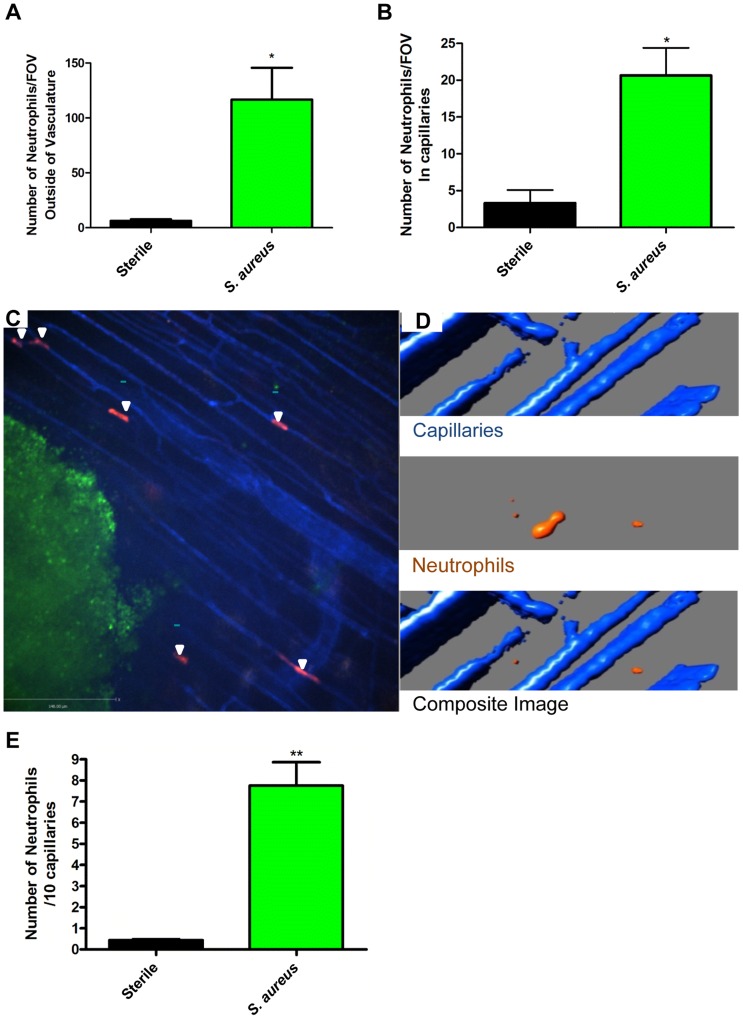
Neutrophil recruitment to capillaries during *S. aureus* infection. 2a: The numbers of neutrophils outside the blood vessels were quantified per FOV at 4× magnification, 2 hours after insertion of the bead into skin tissue. 2b: The number of neutrophils in the capillaries was quantified per FOV, at 4× magnification after insertion of the bead into the skin tissue. N = 3 independent experiments for sterile beads, and 4 independent experiments for *S. aureus* for figures 2a and 2b. Each independent experiment consisted of a single 4× FOV around an agarose bead. 2c: Image of neutrophil recruitment to the capillary microvasculature, taken at 10× magnification. In red are the neutrophils, in green is the *S. aureus* bead, and in blue is the vasculature. White arrows indicate the location of neutrophils in the capillary microvasculature. 2d: 3D reconstruction of a capillary with a neutrophil inside it. Neutrophils are in orange, the capillaries in blue. The upper panel shows the capillaries alone, without any neutrophils. The middle panel shows a neutrophil without any capillaries. The bottom panel shows the overlap of the red and blue channels, with the orange neutrophil inside the blue capillary, and thus not visible. 2e: Number of crawling neutrophils. Neutrophils were determined to be crawling if they moved within the capillary microvasculature, and remained in the FOV for 30 or more seconds. N = 3 independent experiments for sterile beads, 6 independent experiments for *S. aureus* beads. Each N is an average of 4 or more 10 minute videos at 10× FOV from a single mouse. *p<0.05, **p<0.01.

Within the first few minutes, increased numbers of neutrophils could be seen rolling along the side of the vessels adjacent to infected but not non-infected beads. Many of these neutrophils adhered and emigrated. There were significantly more neutrophils emigrating as early as 1 hr after introduction of the beads that contained *S. aureus* when compared with sterile beads (*p* = 0.031). This is quantified in Figure 1c, and illustrated in a series of panels in Figure 1d and Videos S1 and S2. As demonstrated in Video S1, many neutrophils emigrated outside of the vasculature (quantified in Figure 2a) towards the direction of the bead and migrated in that direction.

In every experiment, we identified a subset of neutrophils that were dramatically deformed, elongated like sausages and crawled back and forth in a linear fashion over a distance of a few hundred microns surrounding the bead insertion site. PECAM-1 staining to delineate venules, capillaries, and arterioles in the skin revealed that this population of neutrophils was crawling in the smallest vascular structures, namely capillaries with diameters less than 10 µm (Figure 2b, Figure 2c and Video S3). This behaviour was not noted with sterile beads (Figure 2b). For these experiments, we used the RB6-8C5 anti-Ly6g antibody, which can label Ly6c (a molecule found on monocytes as well as neutrophils). Since monocytes but not neutrophils have previously been described to crawl in capillaries, 1A8, an antibody known to only label Ly6g and thus neutrophil specific [29], was also tested and confirmed that the crawling cells were indeed neutrophils (Supplementary Figure S1). The neutrophils could have been crawling on top of the capillaries using them as scaffolds; however z-stack imaging and 3D image reconstructions clearly demonstrated the neutrophils were inside and not outside the capillaries (Figure 2d).

The neutrophils adhered directly in the mainstream of blood in capillaries, bypassing any rolling event. The neutrophils then immediately began crawling but no neutrophils were ever seen to emigrate from the capillaries in the twelve experiments assessed. In the *S. aureus* infected beads, there were approximately 7.5 neutrophils crawling per 10 viewed capillaries although it was not unusual to see multiple neutrophils in one capillary such that approximately 35–40% of capillaries were laden with crawling neutrophils (Figure 2e, p = 0.007) while sterile beads had fewer than one neutrophil per 10 capillaries.

### Inhibiting Neutrophil Recruitment in Capillaries

**Figure 3:**
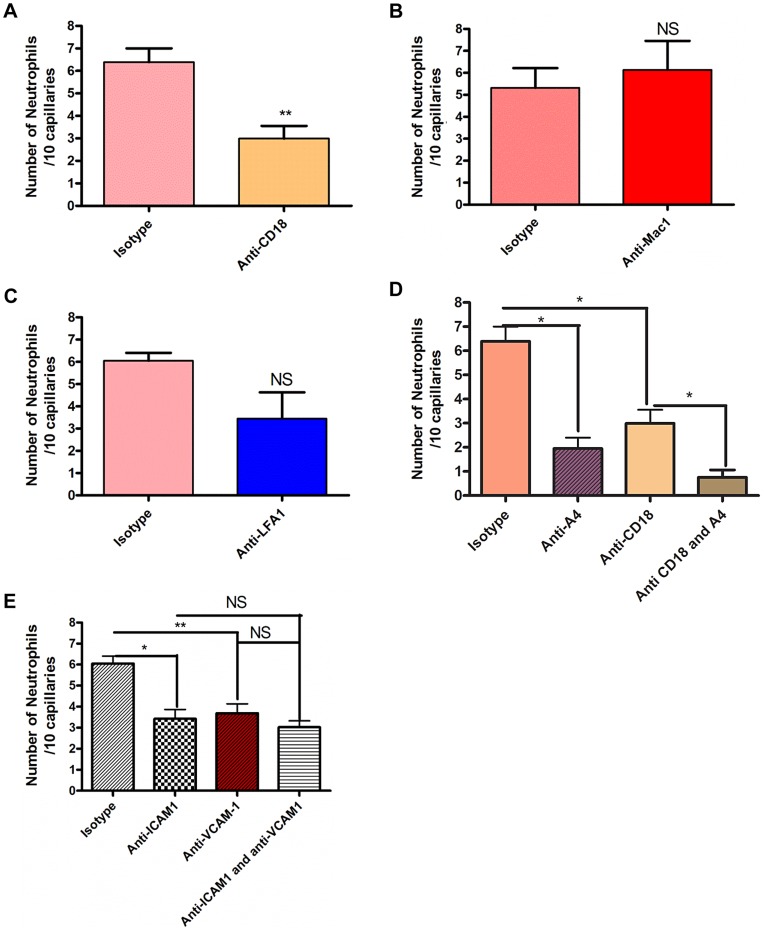
The effect of blocking integrins and their ligands. Two hours after the bead cluster had been inserted into the subcutaneous dorsal skin of C5BL6 mice, various blocking antibodies or isotype controls were injected intravenously. 3a: recruitment of neutrophils to the capillaries over ten minutes after treatment with either 30 µg anti-CD18 antibodies or isotype control antibodies. N = 3 independent experiments for mice given anti-CD18 antibodies, and 4 independent experiments for mice given isotype control antibodies. 3b: recruitment of neutrophils to the capillaries over ten minutes after treatment with either 30 µg anti-Mac 1 antibodies or isotype control antibodies. N = 5 independent experiments for Mac-1 and for isotype control experiments. 3c: recruitment of neutrophils to the capillaries over ten minutes after treatment with either 30 µg anti-LFA1 antibodies or isotype control antibodies. N = 5 independent experiments for mice treated with LFA-1 blocking antibodies and 4 independent experiments for isotype controls. 3d: recruitment of neutrophils to the capillaries over ten minutes after treatment with either CD18 and anti-α_4_ antibodies, CD18 antibodies alone, anti-α_4_ antibodies, or isotype controls. N = 3 independent experiments for all conditions. 3e: recruitment of neutrophils to the capillaries over ten minutes after treatment with 100 µg anti-ICAM-1 antibodies, anti-VCAM-1 antibodies, both antibody treatments or isotype control antibodies. N = 4 independent experiments for all condition states. Each N is an average of 4 or more 10 minute videos at 10× FOV from a single mouse. ** *p*<0.01.

Since neutrophil crawling in capillaries has not been described previously, antibodies to LFA-1 and Mac-1 or to the common β subunit (CD18) of both molecules were used in an attempt to block this event. These are the two major integrins on neutrophils. Rather than pretreatment that could affect other parameters in this process, a very stringent approach of administering antibodies two hours after the infection when crawling was already at its peak was implemented. An antibody to CD18 blocked 60% of the capillary crawling (Figure 3a). Surprisingly the adhesion molecule thought to be dominant for crawling in venules, Mac-1, played no role in crawling in skin capillaries (Figure 3b) while LFA-1 antibody had a trend to reduced crawling (Figure 3c), only blocking the beta chain of the CD18 integrin reached significance (Figure 3a). The drop in recruitment we observed following the blocking of CD18 did not result in a complete inhibition of neutrophil recruitment to the capillaries; close to 40% of neutrophil recruitment remained unexplained. Inhibition of the integrin VLA-4 reduced more than 50% of the recruitment into the capillaries (Figure 3d). Tandem blockade with VLA-4 and the β_2_ integrin antibodies had additive inhibitory effects on the recruitment of neutrophils to the capillaries almost entirely ablating this event (p = 0.0256 Figure 3d). Each antibody intervention was compared to its own IgG isotype control (Figure 3).

We then sought to determine whether the common ligands for these integrins were binding to partner molecules in the capillaries. Our primary targets were ICAM-1 and VCAM-1. These molecules were also targeted because they are known to be expressed on endothelial cells during inflammatory conditions, and have been shown to be upregulated on endothelial cells *in vitro* after stimulation with components of the *S. aureus* cell wall [30]. Blocking ICAM-1 via anti-ICAM-1 antibodies resulted in a significant reduction in the number of neutrophils recruited to the capillaries (*p* = 0.0246, Figure 3e). When VCAM-1 was blocked, there was also significant decrease in the number of recruited neutrophils (*p* = 0.009, Figure 3e). However, blocking both VCAM-1 and ICAM-1 resulted in no significant differences in recruitment compared with either ICAM-1 or VCAM-1 alone (*p* = 0.482, Figure 3e), suggesting that either CD18 or α_4_-integrin adheres to additional ligands besides VCAM-1 and ICAM-1.

### Capillary Perfusion and Cell Death

**Figure 4:**
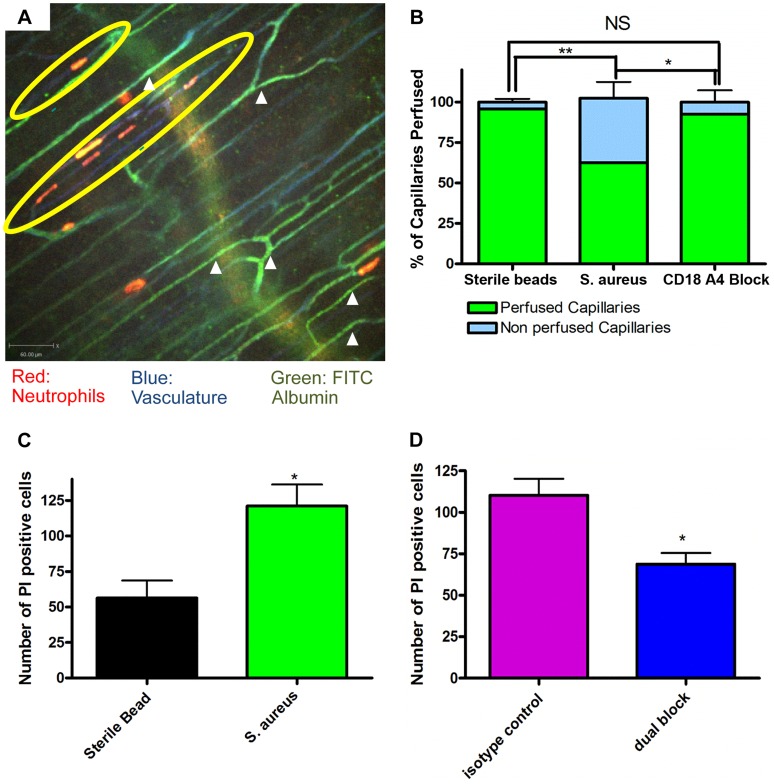
Occlusion of the capillary microvasculature and quantification of cell death following insertion of *S. aureus* bead. 4a: Image of capillary occlusion adjacent to a *S. aureus* bead. Mice were treated with 10 µl at 1.0 g/ml of CD31 and 10 µl of Ly6g at 0.2 g/ml conjugated to PE, as well as *S. aureus* beads, for two hours prior to image acquisition. Mice were then injected with approximately 50 µl of FITC-albumin solution after video acquisition began. Neutrophils are labelled in red, the vasculature in blue, and the flow of blood in green. The *S. aureus* containing bead is located to the right of the image, just out of view. 4b: Graphical representation of occlusion of the capillary microvasculature. Vessels were considered occluded if, 30 seconds after FITC albumin, the vessels were not positive for FITC albumin fluorescence. The numbers of occluded capillaries were quantified as a percentage of the total capillaries present in the FOV. Mice were given blocking antibodies (anti-CD18 and anti-α_4_) 20 minutes before injection of FITC albumin. N = 3 independent experiments for mice with sterile control beads, 4 independent experiments for *S. aureus* beads, and 3 independent experiments for mice given *S. aureus* beads and treated with blocking antibodies. **p<0.01. 4c: The number of propidium iodide positive cells found in FOVs near a bead when comparing sterile and *S. aureus* beads. Data is from multiple fields of view per mouse. N = 3 independent experiments for sterile beads and 5 independent experiments for *S. aureus* beads. 4d: The number of propidium iodide positive cells found in FOVs near an *S. aureus* bead when comparing mice treated with anti-CD18 and anti α_4_ antibodies, and isotype controls. Mice were treated with antibodies at the same time as insertion of *S. aureus* beads. N = 3 for isotype control treatment, N = 4 for the dual block treatment. *p<0.05.

The profound deformation of neutrophils in the capillaries, suggested the potential for vessel occlusion. An intravenous injection of FITC-albumin was used to visualize perfusion through individual blood vessels [31], [32]. All vessels were first labelled with Alexa 647 conjugated to anti-CD31 antibodies (blue). Injection of FITC-albumin turned perfused vessels green (Figure 4a). Almost all of the capillaries in mice treated with sterile beads were perfused with virtually no occlusion of any vessels. Mice treated with *S. aureus* beads had ∼35–40% of the capillaries occluded a value significantly greater than sterile beads (Figure 4b). In general, no neutrophils were lodged inside perfused capillaries (white arrowheads, Figure 4a), and only capillaries, not venules nor arterioles were occluded. When all neutrophil sequestration in capillaries induced by *S. aureus* was prevented with anti-CD18 and anti-α_4_ antibodies, capillary occlusion was significantly reduced (Figure 4b). Capillary occlusion in the antibody-treated *S. aureus* mice was not significantly different from mice treated with the control sterile beads (Figure 4b) suggesting that neutrophil recruitment was causally related to vessel occlusion.

Propidium iodide was used to investigate the degree of cell death in skin. It is worth noting that under non-inflammatory conditions there is always some basal cell death that was not increased by sterile beads (Figure 4c). There was increased cell death with *S. aureus* beads, as compared to sterile beads (Figure 4c). When mice were treated with blocking antibodies (anti-CD18 and anti-α_4_) there was a significant (Figure 4d) reduction in the number of dead cells, compared to isotype control treatment.

**Figure 5:**
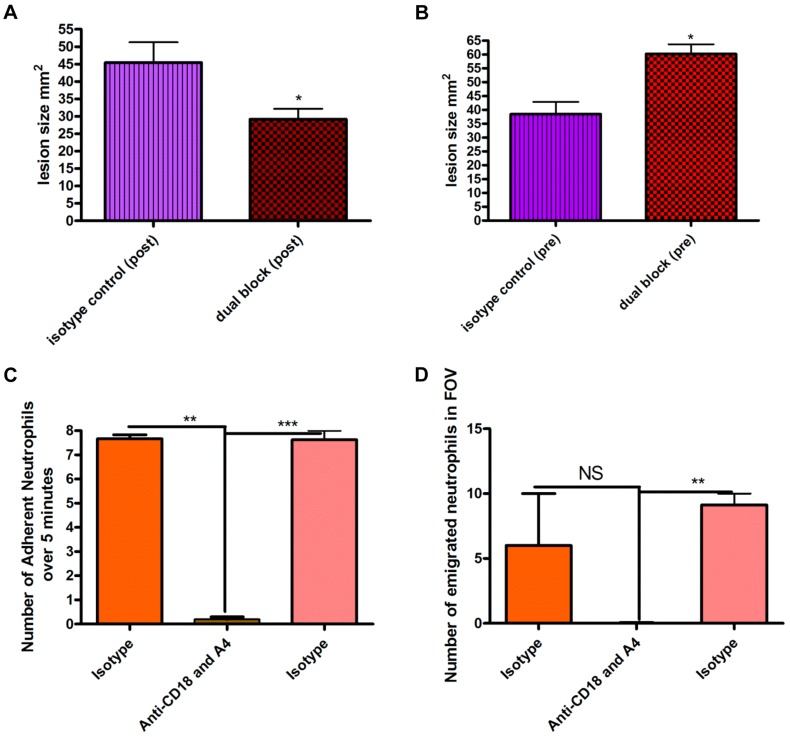
Effect of blocking antibodies on lesion formation 48 hours after bead injection. Beads (approximately 10^6^ CFU) were injected SC into mice. Mice were treated with a cocktail of either isotype control IgG1 and IgG2b antibodies or anti-CD18 and anti-α_4_ antibodies (via tail vein injection) either two hours before or two hours after injection of beads. Tissue was excised 48 hours following infection, and then the lesion was photographed and analyzed using ImageJ software to identify the size in mm^2^. 5a: Effect of blocking antibodies administered two hours after infection by needle injection of *S. aureus* beads. N = 4 independent experiments for isotype control treatment, and 4 independent experiments for dual blocking treatment. Each independent experiment consisted of analysis of a single lesion. 5b: Effect of blocking antibodies injected two hours before infection. N = 4 independent experiments for isotype control treatment and 3 independent experiments for dual block two hours before infection. Each independent experiment consisted of analysis of a single lesion. 5c: the number of adherent neutrophils in a 100 µm stretch of 20–40 µm venule was measured over 5 minutes. Neutrophils were considered adherent if they remained stationary for more than 30 seconds. 5d: the number of emigrated neutrophils in the FOV of a venule was determined over the 5-minute period. Any neutrophil outside of the vasculature was determined to be emigrated. N = 3 independent experiments.

*S. aureus* beads containing 1×10^6^ CFU induced a lesion at 48 hours. Blocking neutrophil recruitment two hours after infection (anti-CD18 and anti-α_4_) reduced lesion size at 48 hours (Figure 5a). Importantly, blocking neutrophil recruitment two hours before infection resulted in increased lesion size compared to mice that only received *S. aureus* beads (Figure 5b), suggesting the initial recruitment of neutrophils is critical. Indeed, the tandem inhibition of both CD18 and anti-α_4_ integrins prevented all neutrophil recruitment to the infectious site, which included neutrophil adhesion (Figure 5c) and emigration in postcapillary venules (Figure 5d). However, administration of antibodies to CD18 and anti-α_4_ integrin two hours after infection did not affect the huge influx of neutrophils into the infectious nidus via postcapillary venules.

## Discussion

Recent research has focused on the complex and often paradoxical role that the innate immune system plays in *S. aureus* infection [2], [11]. Neutrophil recruitment is considered critically important to eradicate *S. aureus* infections, since deficiencies in neutrophil function can impair the host's ability to combat *S. aureus* infections, as demonstrated in both patients and experimental mouse models [33], [34]. However, neutrophil recruitment has also been shown to be highly cytotoxic, causing substantial bystander tissue damage in the process of controlling infections [13], [14]. In this study, we combined the use of spinning disk intravital microscopy and a novel model of *S. aureus* (MRSA USA 300) subcutaneous infection and visualized the complex interaction between neutrophils and this pathogen. Significant recruitment of neutrophils towards localized *S. aureus* infection was noted, despite the use of orders of magnitude fewer bacteria than previously reported. Although many molecules have been described to be released by and allow *S. aureus* to evade detection by neutrophils [35], [36], in this study very robust recruitment of neutrophils occurred. This perhaps highlights the ability of neutrophils to overcome these evasion mechanisms or highlights differences between *in vivo* and *in vitro* results. The latter may reflect the use of *S. aureus* that have upregulated their evasion mechanisms. Therefore, our model permitted systematic examination of bacteria localized around a foreign particle, and analysis of neutrophil behaviour around this nidus of infection.

Although our model corroborates the results of other studies [11], [12], [37]–[40] that demonstrate that neutrophils are actively recruited to the site of *S. aureus* infection via postcapillary venules, we observed a very novel behaviour of neutrophils adhering and subsequently crawling inside the capillaries close to the *S. aureus* beads. The neutrophils were physically deformed taking the shape of the capillaries, and often moved to and fro inside the vessels. Three dimensional reconstruction confirmed that the neutrophils were inside the capillaries. Further evidence supporting that neutrophils were inside the capillaries was their direct inhibitory effect on perfusion of blood through the capillaries. This recruitment occurred in response to the *S. aureus*-infected bead, and not due to sham surgery or the bead alone. It is unlikely that the capillaries functioned as a thoroughfare to deliver neutrophils to the site of infection as no neutrophil was ever observed to emigrate out of these vessels. It is possible that neutrophils were recruited to capillaries to occlude perfusion of the infected tissue and thereby prevent any bacterial dissemination via the vasculature from the initial infectious nidus. The reduced perfusion could also reduce pH making the environment less conducive to survival of the bacteria. Alternatively, the neutrophil sequestration in capillaries could be a defense mechanism induced by *S. aureus* that limits the ability of neutrophils to infiltrate the tissue. Reduced perfusion of tissue could lead to more anaerobic conditions conducive to survival of the pathogen (a facultative anaerobe), and increase tissue damage [17]. Indeed, inhibition of neutrophil recruitment into capillaries resulted in improved perfusion, reduced cell death and significantly reduced lesion size.

One complication with definitively establishing the importance of neutrophil recruitment to the capillaries was the associated inhibition of neutrophil recruitment from the venules. However, allowing neutrophils to infiltrate the tissue in significant numbers over the first 2 hours and then reversing neutrophil recruitment into capillaries reduced some of the pathogenesis associated with the *S. aureus* infection. Presumably, sufficient numbers of neutrophils were recruited to surround the infectious nidus and further neutrophil recruitment was unnecessary and perhaps even toxic. By contrast, preventing all recruitment of neutrophils by pretreating animals with the two anti-integrin antibodies caused greater tissue injury and more bacteria in blood, consistent with the observations by others [35], [36]. In the first few hours of infection, neutrophils are thus absolutely critical to limit bacterial dissemination. It also suggests that when unchecked by neutrophils, the bacteria can cause injury due to their release of many potent toxins. Herein, we demonstrate that in addition to surrounding the infectious nidus via emigration from venules, plugging surrounding capillaries very early might also contribute by preventing bacterial entry into the mainstream of blood. However, this latter event does cause hypoxia, cell death and increased lesion size, so eventually the occlusion of vessels causes pathophysiology and therapeutic intervention would be beneficial.

The presence of α_4_ integrin on neutrophils is controversial [21], [22], [41] and thought to perhaps play a greater role in mouse than human. Although neutrophil recruitment to tissues like muscle, skin and brain are primarily via the CD18 integrin [20], [42], recruitment to tissues like liver or lung can occur independent of this β_2_-integrin. In addition, in both mouse and human, it has been shown that neutrophils can also use the α_4_ integrin VLA-4 in extreme conditions such as systemic infections associated with sepsis. Plasma from a septic human patient could induce the expression of α_4_ integrin on the surface of neutrophils from healthy patients [22]. In addition, this molecule induced functional adhesion to its ligand VCAM-1, although other ligands for α_4_ integrin also exist. In chronic adjuvant arthritis inflammation which was associated with a systemic vasculitis, α_4_ integrin was important in neutrophil recruitment, but VCAM-1 was not involved [43].

Herein, in a localized *S. aureus* infection, the recruitment to the capillaries was mediated by both the β_2_ integrins and the α_4_ integrin. Blocking either Mac1 or LFA-1 alone in our model did not have significant effects on neutrophil recruitment within the capillaries, suggesting that these subunits likely play overlapping roles in the capillaries, and both must be blocked in addition to α_4_ integrin in order to prevent neutrophil recruitment. Perhaps not surprisingly, a role for VCAM-1 was revealed for some of the neutrophil recruitment into capillaries as this molecule is expressed constitutively in murine skin endothelium [44]. The fact that ICAM-1 and VCAM-1 did not completely block recruitment to capillaries suggests that other molecules also are used by the integrins. This is not surprising since these integrins can adhere to many different ligands.

With the discovery of neutrophil crawling in capillaries a number of new issues arise. First, how much capillary occlusion is necessary to cause tissue injury. Although 35–40% of capillaries were occluded in our study, it was impossible to exclude the possibility that injury also occurred due to the proteases and oxidants released by neutrophils that infiltrated the injury site via the post-capillary venules. It is also important to note that all capillary beds are different, raising the importance of imaging the skin when studying skin infections and imaging the liver when studying liver infections. Indeed, CD44 and not integrins are used by neutrophils in the sinusoids of the liver postinfection [45]. Moreover, capillaries of other organs may not have neutrophil crawling or capillary plugging. Finally the reason for why neutrophils crawl in capillaries is unclear. However millions of years of evolutionary pressure directing the fight between this common pathogen and the host may have evolved an important anti-microbial process or an important bacterial evasion mechanism that is still not entirely understood.

In conclusion, in this study a novel neutrophil behaviour has been identified in response to subcutaneous infection due to a virulent strain of *S. aureus*. Using spinning disk confocal microscopy, we noted significant neutrophil recruitment into capillaries surrounding the infectious nidus. We determined that the molecules responsible for this behaviour were the β_2_ and α_4_ integrins, binding in part with ICAM-1 and VCAM-1, and causing occlusion of the capillary microvasculature. Blocking this recruitment at a delayed time point, reduced the malperfusion, cell death and lesion size that developed several days after infection with the *S. aureus* infected bead. As *S. aureus* becomes more resistant to antibiotics, understanding the mechanisms that underlie the pathogenesis of this infection will enhance the likelihood of non-antibiotic therapeutic intervention.

## Materials and Methods

### Mice

C57BL6 male mice (Jackson, Bar Harbour), aged 6–8 weeks were used for all experiments.

### Ethics Statement

All animal protocols were submitted to the animal care committee of the University of Calgary under the protocol number AC12-0222. All animal protocols approved by the animal care committee of the University of Calgary and complied with the Canadian Animal Care guidelines.

### Bacteria

Green fluorescent protein (GFP)-expressing *S. aureus* was made from a previously isolated clinical strain USA300-2406, described previously [46]. Bacteria were grown in 5 ml of Brain Heart Infusion (BHI) media (Becton and Dickenson, Sparks, MD), and were incubated overnight at 37°C. GFP-expressing *S. aureus* (strain USA300-2406) was grown in 20 µg/ml chloramphenicol (EMD Biosciences, La Jolla, CA).

Agarose beads were used to deliver bacteria on a foreign particle, based on an existing model of cystic fibrosis [47]. *S. aureus* were grown overnight in BHI (20 µg/ml chloramphenicol) at 37°C. The next morning, 5 ml of overnight media was mixed with 45 ml of fresh BHI (20 µg/ml chloramphenicol), and grown for a further two hours. *S. aureus* was then centrifuged at 2000 rpm for 10 minutes, and resuspended in 250 µl of 1× phosphate buffered saline (PBS). 10 µl of PBS containing bacteria were serially diluted and plated, to measure CFUs. The remaining PBS was then added to 2.25 ml of liquid 1.5% TSA agar. The TSA/PBS/*S. aureus* solution was then slowly injected into a mixture of 40 ml of mineral oil (Sigma-Aldrich, St Louis, MO) and 400 µl of Tween 20 (Sigma-Aldrich, St Louis, MO), which was gently stirred at 4°C, yielding spherical agarose beads embedded with *S. aureus*. After 15 minutes, the solution was centrifuged at 2000 rpm for 10 minutes. The mineral oil layer was removed, and beads were washed with PBS and resuspended, then spun again at 2000 rpm. This wash step was repeated three times. Beads were then washed in a 100 µl filter, and resuspended with PBS. Beads were stored at 4°C for up to 6 days. Plating of 1–6 day old beads on fresh agar showed no loss of CFU within this timeframe. For sterile beads (control), bacteria were replaced with 2 µl of Fluoresbrite plan yg 1.0 micron microspheres (Polysciences, Warrington, PA)

#### Imaging of the murine skin tissue

Male C57B6 mice were anesthetised with 200 mg/kg ketamine (Rogar/STB, London, ON) and 10 mg/kg xylosine chloride (MTC Pharmaceuticals, Cambridge, ON) injected intra-peritoneally 20 minutes prior to surgery. Skin preparation was conducted as previously described [46]. Briefly, mice were kept at body temperature using a heating pad and a jugular catheter was inserted to maintain anesthesia and deliver systemic treatments. The dorsal flank skin was exteriorized on the right flank. A 4-0 suture (Ethicon, Markham, ON) was used to pierce and thread through the skin tissue at the edge of the exteriorized skin tissue. Three bead clusters, of a diameter between 250 and 350 µm were selected and inserted into the exteriorized subcutaneous tissue (underneath the the connective tissue of the skin) to a maximum depth of 50 µm, a minimum of 5 mm away from the edge of the tissue. The tissue was then covered with a cover slip (VWR, Radnor, PA), which was connected to the plastic board with high vacuum grease (Dow Corning, Midland, MI). Superfusion buffer was then perfused across the exteriorized skin tissue. Approximately 150 ml of superfusion solution (NaCl 7.70 g/L, KCl 0.350 g/L, CaCl_2_ 0.222 g/L, MgSO_4_ 0.144 g/L, NaHCO_3_ 1.68 g/L) at a pH of 7.4 (±0.10) and 37°C was used to keep the skin moist and devoid of any untoward inflammation when control beads were administered. Mice were imaged for up to 4 hours after being anesthetized with 10 mg/kg xylosine chloride/ketamine.

##### Spinning disk intravital microscopy

Spinning disk confocal microscopy was performed using an Olympus BX51 (Olympus, Center Valley, PA) upright microscope. The microscope used a confocal light path (WaveFx, Quorum) based on a modified Yokogawa CSU-10 head (Yokogawa Electric Corporation). Laser excitation at 488, 561 and 647 was used in rapid succession and fluorescence in green, red and far red channels was visualized with long pass filters (Semrock). Exposure time and sensitivity setting were uniformly maintained for each set of experiments. For imaging, velocity acquisition software (Improvision Inc. Lexington, KY) was used to drive the microscope. A 512×512 pixel back thinned EMCCD camera (C9100-13 Hamamatsu) was used for fluorescence detection. Mice were imaged using a 4×/0.16 air objective (Olympus, Center Valley, PA). Some mice were also imaged as required following 2 hours of infection with a 10×/0.30 numerical aperture air objective (Olympus, Center Valley, PA).

##### Labelling antibodies

Mice were injected with 10 µl of anti-Ly6G (clone RB6-8C5) conjugated with PE (Ebioscience, San Diego, CA), at a concentration of 0.2 mg/ml. This concentration of a single bolus injection of antibody is sufficient to allow us to image neutrophils for up to 4 hours without excessive bleaching of signal while in no way affecting neutrophil recruitment parameters [48]. In experiments where propidium iodide was used, mice were injected either with 10 µl anti-Ly6g (clone RB6-8C5) conjugated with Alexa680 (Ebioscience, San Diego, CA) at a concentration of 0.2 mg/ml, or 10 µl of anti-Ly6g (clone 1A8) conjugated with PE (BioLegend, San Diego, CA) at a concentration of 0.2 mg/ml. The vasculature was also labelled with 10 µl of CD31 (Clone 390, Ebioscience, San Diego, CA) conjugated to Alexa647 (Molecular Probes, Eugene, OR), at 1.0 mg/ml. CD31 was conjugated in house to Alexa647 with a labeling kit (Molecular Probes, Eugene, OR).

##### Lesion size experiments

Bead clusters (between 250–350 µm) were selected and isolated in 100 µl of saline. Three bead clusters were vortexed for approximately 60 seconds and then serially plated on BHI agarose (Becton and Dickenson, Sparks, MD) plates with 20 µg/ml chloramphenicol. Mice were temporarily anesthetized by isoflouride and a single bead cluster per mouse was then injected subcutaneously, in the same region as the beads were placed in previous experiments, using a 16 gauge needle (BD, Franklin Lakes, NJ). Mice were then returned to their cages. Mice were sacrificed 48 hours after infection, and skin (epidermis to subcutaneous) was harvested around the area of the bead injection (up to 2×2 cm^2^.) The tissue was photographed and measured with a ruler and then placed in 10% neutral buffered formalin (EMD Chemicals, Gibbstown, NJ) for storage. Preliminary experiments revealed no lesion with sterile beads while lesion occurred when *S. aureus* was injected without beads, suggesting it is the *S. aureus* and not the beads *per se* that cause the lesions.

### Reagents

#### Blocking antibodies

Blocking antibodies were administered intravenously in saline in most imaging experiments two hours following introduction of the bacteria into the host. Quantification of the effect of blocking antibodies was performed 20 minutes after injection. In experiments using propidium iodide, blocking antibodies were administered 5 minutes before the introduction of S. aureus. In lesion studies, blocking antibodies were administered two hours before or after introduction of bacteria into the host. Isotype control antibodies were: IgG2b at 100 µl and 50 µl volumes at a concentration of 1.0 mg/ml (Pharmigen, San Diego, CA), IgG2a, at 100 µl and 30 µl, at a concentration of 1.0 mg/ml, (Pharmigen, San Diego, CA) and IgG1, at 30 µl at a concentration of 1.0 mg/ml (Pharmigen, San Diego, CA). For blocking the β_2_ integrins, 30 µl of the monoclonal anti-CD18 antibody clone game46 was used at a concentration of 1.0 g/ml (Pharmigen, San Diego, CA). For blocking LFA1, 20 µl of the monoclonal anti-CD11a antibody clone M17/4 was used at a concentration of 1.0 mg/ml (Ebioscience, San Diego, CA). For blocking Mac1, 20 µl of the monoclonal anti-CD11b clone M1/70 at a concentration of 1.0 mg/ml (Ebioscience, San Diego, CA). For blocking VLA-4, 50 µl of the anti-α_4_ integrin antibody clone r1-2 was used at 1.0 mg/ml (Pharmigen, San Diego, CA). For blocking ICAM-1, 100 µl of the monoclonal anti-ICAM-1 antibody YN1/1.7.4 at a concentration of 1.0 mg/ml was used (Ebioscience, San Diego CA). For blocking VCAM-1, 50 µl of the monoclonal and anti VCAM-1 antibody clone 429 at 1.0 mg/ml was used (Ebioscience, San Diego CA). All of these concentrations were derived from previous experiments where optimal doses were established [49].

#### FITC albumin experiments

FITC albumin was prepared in house. Bovine fluorescein isothyiocyanate albumin (Sigma-Aldrich, St Louis, MO) was diluted to 5 mg/ml. Fifty µl was injected IV with saline into anesthetized mice, with exteriorized skin tissue. Mice skin preps were imaged as the FITC albumin was being injected.

#### Propidium iodide experiments

Fifty µl of 2 µM propidium iodide was superfused over the exteriorized tissue. Cell death was measured by counting the number of PI positive cells present. Mice were anesthetized with ketamine/xylosine and the skin tissue was exteriorized. Mice were then injected with CD31 and Ly6g labelling antibodies, as well as isotype controls or dual blocking antibodies intravenously at approximately the same time as the bead was inserted. Mice were treated with propidium iodide at the 2 hour timepoint. Propidium iodide was superfused across the tissue for 30 seconds, and then rinsed off with superfusion buffer. Subsequently images were collected for up to 5 minutes. The number of propidium iodide positive cells was quantified per field of view within a 500 um concentric ring around the bead. To ensure unbiased data collection, 4 images were taken, one east, one west, one north and one south and directly adjacent to the bead cluster in every mouse. The total number of propidium iodide positive cells was averaged over the fields of view per mouse.

### Analysis

Images were analyzed by removing light collected from the 488 and 561 and 649 nanometer channels of the spinning disc confocal microscope. The contrast and brightness used to analyze data was held constant for analysis of each set of experiments. The number of neutrophils at 30, 60, 90, and 120 minutes was counted by using the point tool function of Volocity (Perkin-Elemer, Waltham, MA). For analysis of location of neutrophils, the 649 channel was used to examine the vasculature. Neutrophils that co-localized with CD31 labelled vessels were determined to be inside the capillaries if the vessels did not exceed 10 µm in width. Neutrophils both inside and outside the capillaries were counted using the point tool function of Volocity.

#### 10× magnification of neutrophil recruitment within the capillaries

Ten minute videos were used to quantify the number of neutrophils in the capillaries. All capillaries within a 500 µm concentric ring of the S. aureus bead were examined. An average of 14 capillaries were imaged per mouse. Over 10 minutes, the number of neutrophils that were adherent or crawling within the microvasculature was quantified. Neutrophils were classified as adherent if they remained stationary within the FOV for thirty or more seconds. Neutrophils were classified as crawling if they remained within the FOV for thirty or more seconds, and moved at time of data collection. If a neutrophil crawled, it was not counted as adherent. The definitions for these parameters were based on adhesion and crawling measurements in the post-capillary venules, as described previously [20]. The number of neutrophils overall (crawling+adherent) and the individual parameters of crawling and adhering neutrophils were then normalized by dividing the total number of neutrophils by the total number of capillaries, then multiplying by 10. To quantify behaviour, multiple videos (minimum four) were analyzed, and then averaged per mouse.

#### 3D image generation of neutrophils within the capillaries

For some experiments, Z-stacks were taken during imaging of the skin at 10× magnification after two hours of imaging. Z-stacks consisted of 10 slices, taken 2 µm apart. Z-stacks were imaged for 10 minutes. The images were then processed using Volocity software (Perkin-Elemer, Waltham, MA).

#### Measurement of venule parameters

The parameters of adhesion and emigration were also measured in venules within 500 µm of the bead. The number of adherent neutrophils within the 100 µm section of the venule was quantified over 5 minutes of the video. Neutrophils were defined as adherent if they remained stationary for 30 or more seconds. The number of neutrophils per FOV external to any vessel at the 5 minute timepoint of the video in question was defined as the number of emigrated neutrophils.

#### Lesion size experiments

Photographs were analyzed using imageJ (National Institutes of Health, USA) to quantify the size of the lesion. The lesion was traced and the size in pixels was determined and then divided by the number of pixels required for single millimeter squared, to determine the overall area of the lesion.

#### FITC albumin experiments

The image was analyzed 30 seconds after FITC albumin became visible within the vasculature. The number of capillaries were quantified and then scored as either perfused (green) or not perfused (not green) at the 30 second timepoint. This number was then converted into a percentage of the total capillaries quantified.

### Statistical tests

Data was analyzed using the Students t-tests to compare two different conditions. When more than one comparison was made in the same graph, a bonferroni correction was used to correct for false positives. When three variables were all compared with one another in the same graph, a one-way analysis of variance (ANOVA) with a Bonferroni correction was used. All statistical analysis was performed using the statistical software GraphPad prism 4, version 4.03 (GraphPad Software Inc., La Jolla, CA).

## Supporting Information

Figure S1 Comparison of the recruitment of cells labelled by the anti-Ly6g antibody clones RB6 8C5 and 1A8. Mice were treated with 10 µl at 0.2 mg/ml of either antibody conjugated to PE, as well as 10 µl at 1.0 mg/ml anti-CD31 conjugated to Alexa647, IV. S1a: recruitment of cells labelled with either RB6 8C5 or 1A8. The number of neutrophils was quantified at 30, 60, 90 and 120 minutes post-insertion of the bead into the skin tissue. S1b: recruitment of neutrophils to the capillaries over ten minutes after labelling with either anti-Ly6g antibody clone RB6 8C5 or anti-Ly6g antibody clone 1A8 control antibodies. N = 11 independent experiments for the anti-Ly6g antibody clone RB6 8C5 and 4 independent experiments for the anti-Ly6g antibody clone 1A8. Independent experiments consisted of one 4× FOV for Figure S1a and four or more 10× FOV's (averaged) for figures S1b, c and d. In 10× FOVs, the number of neutrophils per 10 capillaries was averaged. 1c: Comparison of the number of crawling cells identified using the anti-Ly6g antibody clone RB6 8C5 and the anti-Ly6g antibody clone 1A8. S1d: Comparison of the number of adherent cells identified using the anti-Ly6g antibody clone RB6 8C5 and the anti-Ly6g antibody clone 1A8. Differences between the clones were non-significant at *p*<0.05. N = 6 independent experiments for the anti-Ly6g antibody clone RB6 8C5 and 4 independent experiments for the anti-Ly6g antibody clone 1A8. ** *p*<0.01.
(PDF)

Video S1 Recruitment of neutrophils to a bead containing GFP-expressing *S. aureus*. Spinning disk microscopy was performed to collect a time lapse video over 2 hours of a bead cluster in the mouse skin model. Neutrophils (red) are recruited and crawl in large numbers over 2 hours towards the *S. aureus* stimulus (green).
(ZIP)

Video S2 Recruitment of neutrophils to a sterile bead containing fluorescent microspheres. Spinning disk microscopy was performed to collect a time lapse video over 2 hours of a bead cluster in the mouse skin model. Neutrophils (red) move in proximity to the bead containing fluorescent microspheres (green), but are recruited in lower numbers than with beads containing *S. aureus*.
(ZIP)

Video S3 Recruitment of neutrophils in the capillaries. Spinning disk microscopy was performed to collect a time lapse video 2 hours after infection. Neutrophils (red) adhere and crawl within the small vessels of the vasculature (blue), moving with and against flow of blood. This behaviour occurs within 500 µm of the *S. aureus* containing bead (green).
(ZIP)
