# Supplementary figures and images for: Neutrophil Crawling in Capillaries; A Novel Immune Response to Staphylococcus aureus
Source: PLoS Pathog. 2014 Oct 9;10(10):e1004379. doi: 10.1371/journal.ppat.1004379 (PMC4192594; doi:10.1371/journal.ppat.1004379)

**SA**

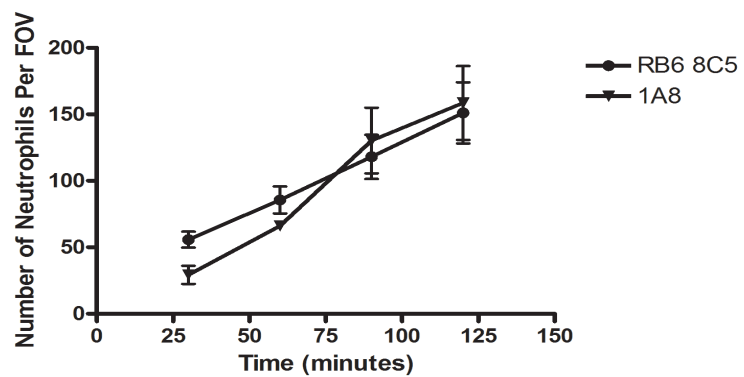

**SB**

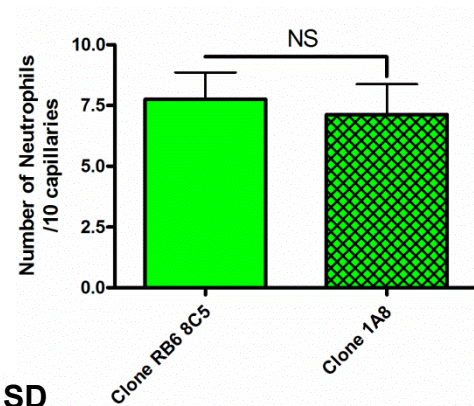

**SC**

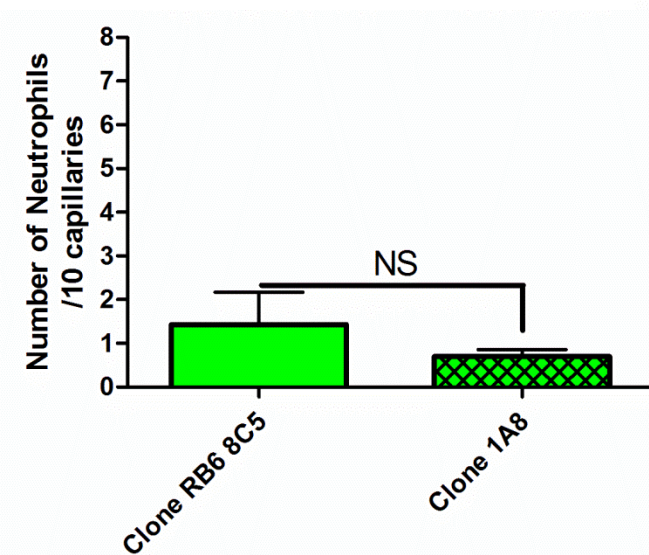

**SD**

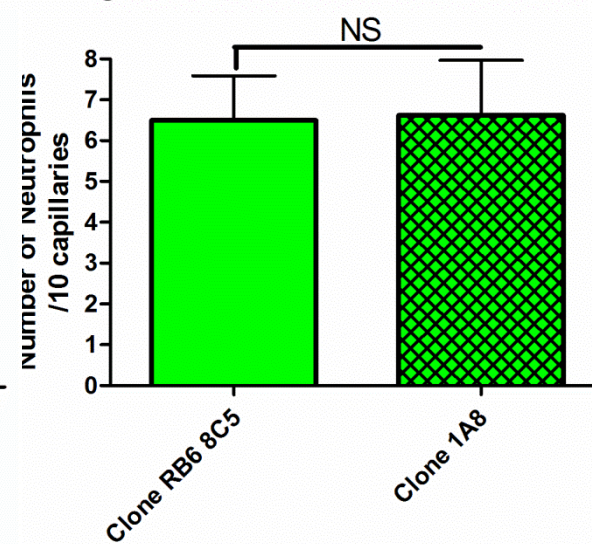

Supplement: Figure S1 — Comparison of the recruitment of cells labelled by the anti-Ly6g antibody clones RB6 8C5 and 1A8. Mice were treated with 10 µl at 0.2 mg/ml of either antibody conjugated to PE, as well as 10 µl at 1.0 mg/ml anti-CD31 conjugated to Alexa647, IV. S1a: recruitment of cells labelled with either RB6 8C5 or 1A8. The number of neutrophils was quantified at 30, 60, 90 and 120 minutes post-insertion of the bead into the skin tissue. S1b: recruitment of neutrophils to the capillaries over ten minutes after labelling with either anti-Ly6g antibody clone RB6 8C5 or anti-Ly6g antibody clone 1A8 control antibodies. N = 11 independent experiments for the anti-Ly6g antibody clone RB6 8C5 and 4 independent experiments for the anti-Ly6g antibody clone 1A8. Independent experiments consisted of one 4× FOV for Figure S1a and four or more 10× FOV's (averaged) for figures S1b, c and d. In 10× FOVs, the number of neutrophils per 10 capillaries was averaged. 1c: Comparison of the number of crawling cells identified using the anti-Ly6g antibody clone RB6 8C5 and the anti-Ly6g antibody clone 1A8. S1d: Comparison of the number of adherent cells identified using the anti-Ly6g antibody clone RB6 8C5 and the anti-Ly6g antibody clone 1A8. Differences between the clones were non-significant at p<0.05. N = 6 independent experiments for the anti-Ly6g antibody clone RB6 8C5 and 4 independent experiments for the anti-Ly6g antibody clone 1A8. ** p<0.01. (PDF) [file ppat.1004379.s001.pdf]
